# Oral [60]fullerene reduces neuroinflammation to alleviate Parkinson's disease via regulating gut microbiome

**DOI:** 10.7150/thno.85711

**Published:** 2023-09-04

**Authors:** Xue Li, Ruijun Deng, Jie Li, Hui Li, Zhe Xu, Lei Zhang, Linyin Feng, Chunying Shu, Mingming Zhen, Chunru Wang

**Affiliations:** 1 Beijing National Laboratory for Molecular Sciences, Key Laboratory of Molecular Nanostructure and Nanotechnology, CAS Research/Education Center for Excellence in Molecular Sciences, Institute of Chemistry, Chinese Academy of Sciences, Beijing 100190, China.; 2 University of Chinese Academy of Sciences, Beijing 100049, China.; 3 Beijing Fullcan Biotechnology Co., Ltd., Beijing, 100085, China.; 4 Chifeng Fullcan Biotechnology Co., Ltd., Inner Mongolia, 024099, China.; 5 Shanghai Institute of Materia Medica, Chinese Academy of Sciences, Shanghai 201203, China.

**Keywords:** Oral fullerene, Parkinson's disease, Gut microbiota, Neuroinflammation, Neurodegeneration

## Abstract

Neuroinflammation is considered to drive the pathogenic process of neuronal degeneration in Parkinson's disease (PD). However, effective anti-neuroinflammation therapeutics for PD still remain dissatisfactory. Here we explore a robust therapeutic strategy for PD using anti-neuroinflammatory fullerenes.

**Methods:** Oral fullerene was prepared by a ball-milling method. 1-methyl-4-phenyl-1,2,3,6-tetrahydropyridine (MPTP)-induced PD mouse model was used to investigate the therapeutic effects and mechanisms of it. The gut microenvironment was evaluated by 16S rRNA gene sequencing, gas chromatography-mass spectrometry, quantitative polymerase chain reaction (Q-PCR), and western blot (WB). The neuroinflammation and neurodegeneration were evaluated by pathological analysis, Elisa kits, transmission electron microscopy, Q-PCR, WB and so on. Toxicity was assessed by weight, blood test and hematoxylin-eosin (HE) staining.

**Results:** Oral fullerene therapeutic system that dissolved [60]fullerene into olive oil (abbreviated as OFO) was dexterously designed, which could reduce neuroinflammation via regulating the diversity of gut microbiome, increasing the contents of short chain fatty acids (SCFAs) and recovering the integrity of gut barrier. Accordingly, the reduction of neuroinflammation prevented dopaminergic neuronal degeneration. And thus, OFO significantly ameliorated motor deficits and fundamentally reversed dopamine (DA) loss in MPTP-induced PD mice. Of note, OFO exhibited low toxicity towards the living body.

**Conclusion:** Our findings suggest that OFO is a safe-to-use, easy-to-apply, and prospective candidate for PD treatment in clinic, opening a therapeutic window for neuroinflammation-triggered neurodegeneration.

## Introduction

Parkinson's disease (PD) is the second most common neurodegenerative disease after Alzheimer's disease, which is caused by dopaminergic neuronal degeneration in substantia nigra and the resultant deficits of dopamine (DA) in the striatum 1, 2. Studies increasingly support that neuroinflammation is primarily implicated in the progression of PD 3, 4. It contributes to the deleterious cascade of events leading to neuronal degeneration, including microglial cells activation, proinflammatory cytokines increase and neuronal apoptosis 5-7. However, it is challenging for the present medications to resolve the neuroinflammation in neurodegenerative disorders due to the presence of blood brain barrier (BBB) and their potential risk 8, 9.

The increasing studies highlighted that the gut microbiota is closely involved in regulating neuroinflammation 10, 11. The dysbiosis of gut commensals would jeopardize intestinal homeostasis and gut barrier integrity, commonly inducing inflammatory microenvironment, thereby resulting in microglia activation and neuroinflammation 12-15. Particularly, short chain fatty acids (SCFAs, the primary bacterial metabolites) might influence neuroinflammation via direct humoral effects, indirect hormonal and immune pathways 16, 17. Therefore, gut microbiota intervention could be a potential strategy to reduce neuroinflammation for PD treatment.

**Scheme 1:**
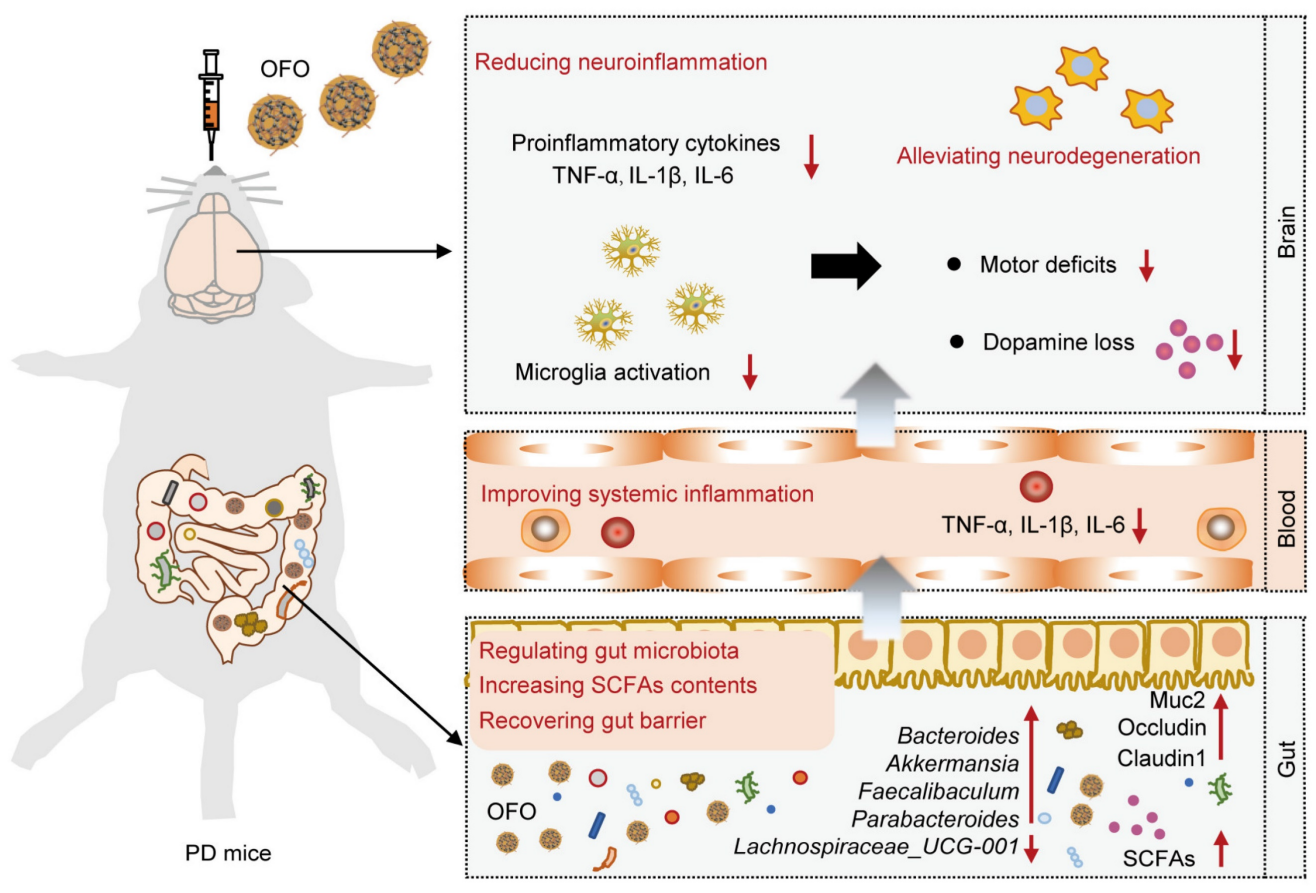
** Schematic illustration of the mechanism for treating PD by OFO.** After orally administrated by PD mice, OFO primarily accumulated in gastrointestinal tract, regulated gut microbiota with increased SCFAs contents and recovered gut barrier, improved systemic inflammation, and then reduced neuroinflammation, thereby alleviating neurodegeneration for treating PD.

Here, we proposed an effective avenue for reducing neuroinflammation by oral [60]fullerene dissolved into olive oil (OFO for short). We demonstrated that OFO is primarily distributed in gastrointestinal tract and regulated gut microbiome with the increase of beneficial bacteria, promotion of SCFAs production, and recovery of gut barrier. We also found that OFO reduced colonic and systemic inflammation, resulting in deactivating microglia activation and decreasing brain proinflammatory cytokines release. Accordingly, OFO alleviated neuronal degeneration by inhibiting mitochondria-mediated neuronal apoptosis. Therefore, OFO exerted excellent therapeutic effects on PD, improving the motor dysfunction and reversing dopamine (DA) deficits (**Scheme 1**). Altogether, our work highlighted a promising therapeutic candidate of oral fullerene for neuroinflammation-related neurodegenerative disorders.

## Results

### The preparation and characterization of OFO

**Figure 1:**
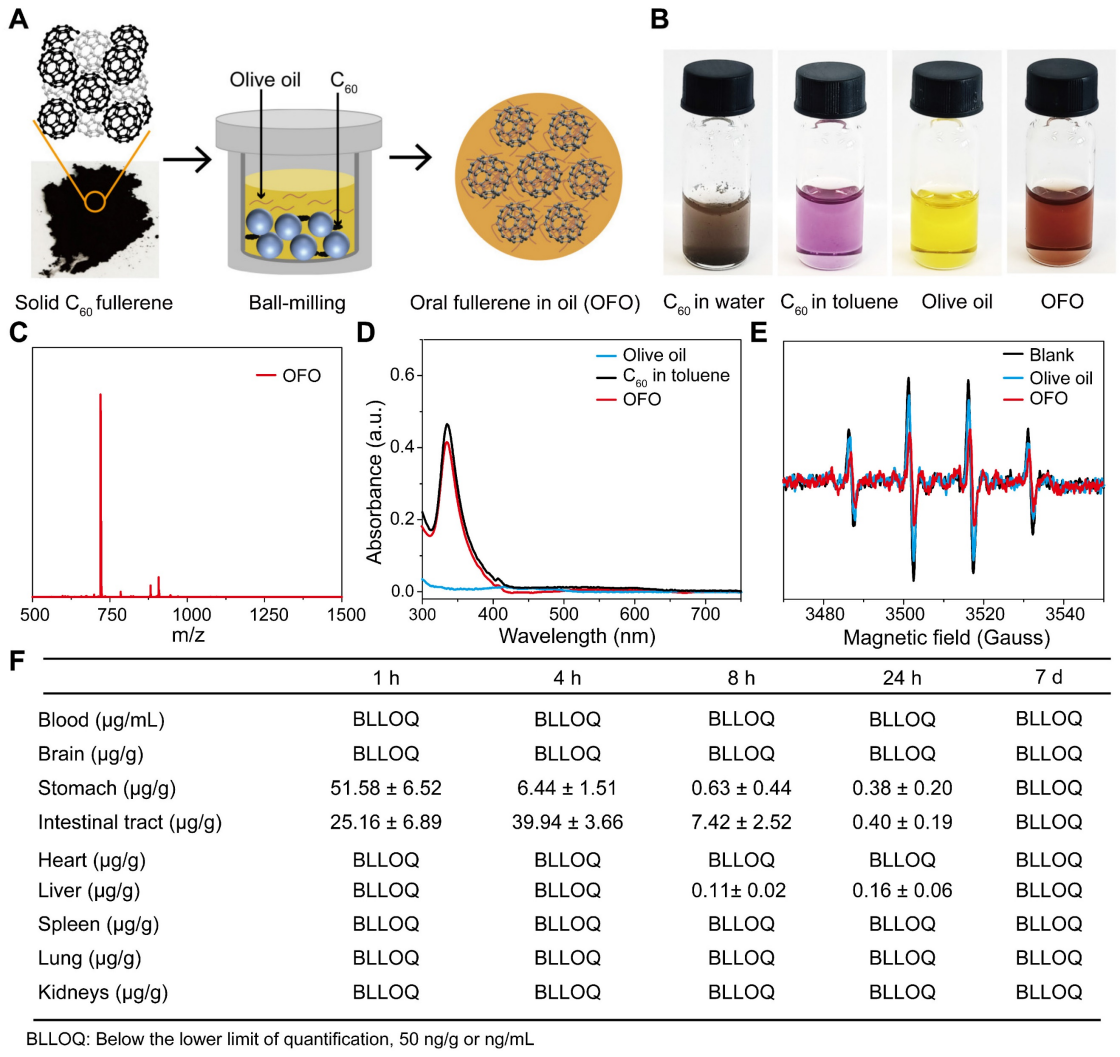
**Preparation and characterization of OFO.** (**A**) The preparation of OFO. (**B**) Photos of C_60_ in water, C_60_ in toluene, olive oil and OFO. (**C**) MALDI-TOF MS of OFO. (**D**) UV-vis absorption spectra of olive oil, C_60_ in toluene (6.25 μg/mL), and OFO (5.80 μg/mL). (**E**) Scavenging •OH abilities of olive oil and OFO at 12.5 μg/mL were assessed by EPR. (**F**) The biodistribution of OFO measured by LC-MS.

OFO was obtained by mixing [60]fullerene with olive oil via a ball-milling method. Briefly, C_60_ fullerene was first separated and purified until the purity reached to 99.9% (Figure S1A-B). Then, solid C_60_ was dissolved in the olive oil and ball-milled at ambient temperature in the dark, forming to a brown oil solution (Figure 1A). Different from suspending in water with insoluble particulate matter, C_60_ formed a clear and transparent solution in olive oil similar to that in toluene solution (Figure 1B). We performed the matrix-assisted laser desorption ionization time-of-flight mass spectrometry (MALDI-TOF MS) to determine the type of fullerene in OFO and found there was a mass-to-charge ratio (m/z) of 720 (Figure 1C), suggesting the pristine structure of C_60_ in OFO. In addition, the characteristic absorption peak at about 330 nm in the ultraviolet-visible (UV-Vis) spectrum of OFO was almost the same as C_60_ in toluene, indicating the integrity of carbon cages of C_60_ in OFO (Figure 1D). The concentration of C_60_ in OFO was determined with 0.87 mg/mL by high performance liquid chromatography (HPLC). Further, we assessed the ability of OFO for scavenging the hydroxyl radical (•OH) using electron paramagnetic resonance (EPR) spectrum. It revealed that approximately 52.8 ± 4.6% of the generated •OH was scavenged by OFO at a concentration of 12.5 μg/mL compared with the blank control, which is much higher than that of olive oil (18.1 ± 8.4%) (Figure 1E).

Then, we quantitatively measured the content of C_60_ in tissues and organs using the liquid chromatography-mass spectrometry (LC-MS) (**Figure 1F**). We observed that OFO rarely accumulated into brain, blood and other main organs, such as heart, spleen, lung, kidneys, and only the trace amount of C_60_ in liver at 24 h was examined, possibly resulting from the phagocytosis by microfold cell 18. On the contrary, C_60_ was primarily distributed in the contents of stomach and intestine after oral administration. Particularly, there was bare accumulation of C_60_ in the living body after 7 days. These results demonstrated that OFO mainly stayed in the gastrointestinal tract and few retained in the mice.

### OFO regulates gut microbiota, elevates SCFAs contents, and recovers gut barrier

**Figure 2:**
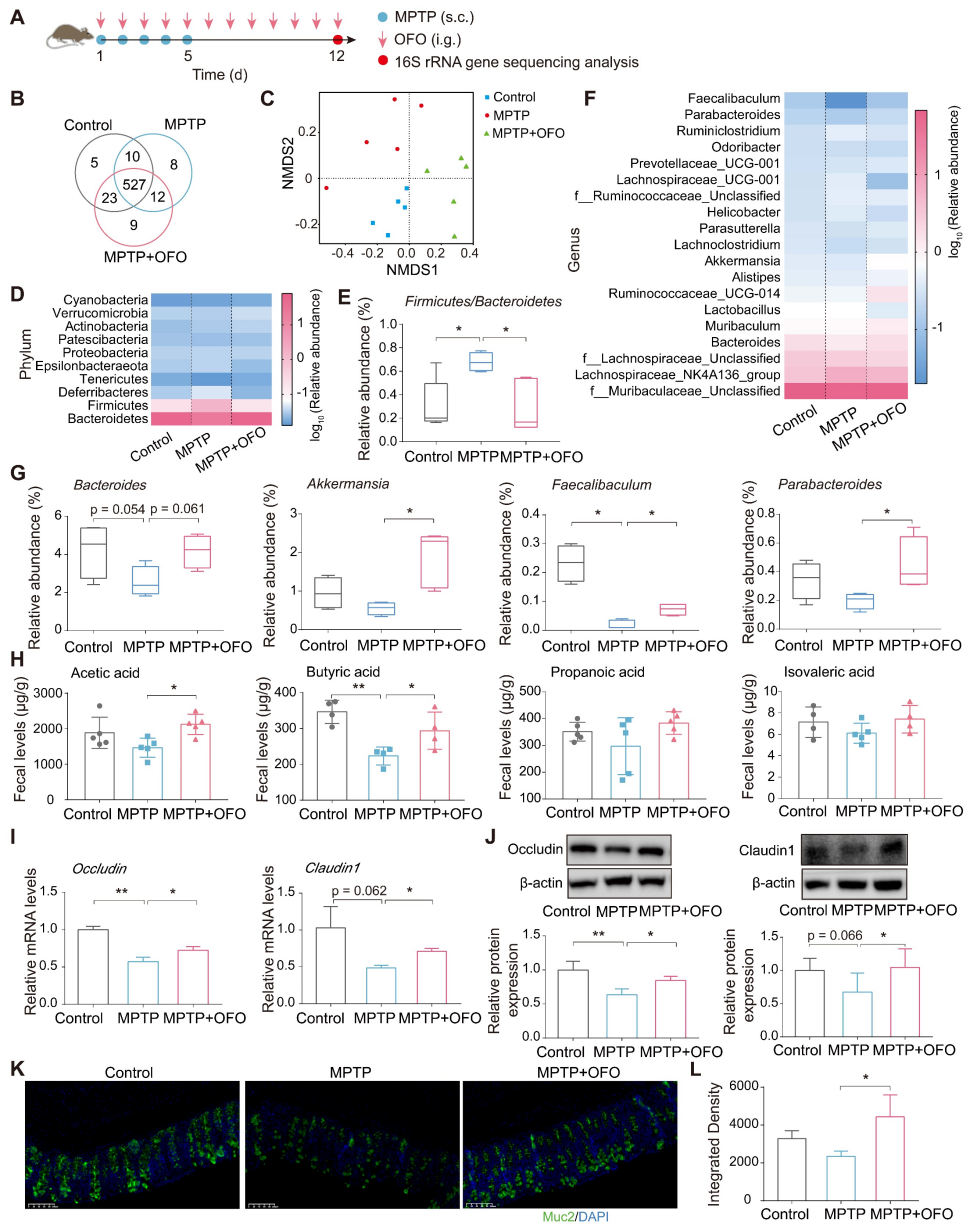
** OFO impaired gut disturbance in MPTP-induced PD mice.** (**A**) The flow chart of gut microbiota analysis. (**B**) Venn diagram of the gut microbiota. (**C**) Analysis of the variances among the microbiol communities by NMDS. (**D**) The relative abundance of the major microbiota at the phylum level. (**E**) The *Firmicutes/Bacteroidetes* ratio in different groups. (**F**) The relative abundance of the major microbiota at the genus level. (**G**) Relative abundance of the *Bacteroides, Akkermansia, Faecalibaculum, and Parabacteroides* at genus level. (**H**) The contents of acetic acid, butyric acid, propanoic acid, and isovaleric acid in feces. (**I**) Relative mRNA levels of *occludin* and* claudin1* in colon tissues analyzed by Q-PCR. (**J**) Relative protein expression of occludin and claudin1 in colon tissues determined by WB. (**K**) Fluorescence microscopy of Muc2 (green) and DAPI (4′, 6-diamidino-2-phenylindole, blue) in colon. Scale bars represent 100 μm. (**L**) Quantitative analysis of immunofluorescence of Muc2. Data are the mean ± S.D. n = 3-7 in each group. *p < 0.05 and **p < 0.01. Data were analyzed using one-way ANOVA with LSD test or Games-Howell test by SPSS.

Considering the abovementioned accumulation of OFO in intestinal tract, we explored the impact of OFO on gut microenvironment including microbiota and its metabolites. First, 16S rRNA gene sequencing in MPTP-induced PD mouse models was conducted (**Figure 2A**). According to the 97% sequence similarity identification criterion, about 594 operation taxonomy units (OTUs) were identified and analyzed. The Venn diagram described that up to 527 species shared among the control, MPTP, and MPTP+OFO groups (**Figure 2B**). Then, the analysis of alpha diversity using Chao1 index and Shannon index revealed that OFO increased the richness and diversity of the gut microbiota (**Figure S2A-B**). And OFO treatment induced a distinct clustering of microbial community structure with stronger resemblance to the controls than that of MPTP group, as shown by principal component analysis (PCA), principal coordinates analysis (PCoA), and nonmetric multidimensional scaling analysis (NMDS) (known as beta diversity, **Figure S3C-D** and **Figure 2C**). Importantly, we exploited the analysis of microbiota composition at different taxonomic levels. At the phylum level, the two most predominant phyla were *Bacteroidetes* and *Firmicutes* (**Figure 2D**), whose ratio played a critical role in maintaining intestinal homeostasis 19. Particularly, MPTP-induced PD mice had a higher *Firmicutes/Bacteroidetes* ratio, whereas OFO reversed this abnormity (**Figure 2E**). At the genus level, OFO showed enriching effects on *Bacteroides, Akkermansia, Faecalibaculum,* and *Parabacteroides* (**Figure 2F-G**), which were beneficial to the gut structure and function 20-22. The level of* Lachnospiraceae_UCG-001*, which was considered to be the key intestinal bacteria for the formation of cognitive impairment 23, was abnormally increased in PD mice, and OFO reduced it (**Figure S3**). In addition, we determined the amounts of SCFAs by gas chromatography-mass spectrometry (GC-MS). It revealed that OFO observably increased the levels of the major SCFAs, such as acetic acid and butyric acid, in feces of MPTP-induced PD mice. Further, OFO exerted a similar enriching effect on propanoic acid and isovaleric acid, though the increase was not significant statistically (**Figure 2H**). Importantly, the relative correlation between SCFAs contents and relative abundance of individual genera was analyzed (**Figure S4**), confirming that most of the elevated genera by OFO was positively correlated with the primary SCFAs and the lowered one was negatively correlated. To assess the effects of OFO on gut barrier integrity, we examined the expressions of the primary tight junction proteins of occludin and claudin1, and the critical physical network barrier of Muc2 in colon of MPTP-induced PD mice by Q-PCR, WB, and immunofluorescence analysis. It was demonstrated that OFO elevated the mRNA and protein expressions of occludin and claudin1, which were lowered in MPTP-induced PD mice (**Figure 2I-J**). And OFO also increased the fluorescent intensities of Muc2 in colon of PD mice, indicating the enhancement of gut barrier (**Figure 2K-L**). All these results unraveled that OFO regulated gut microbiome, promoted SCFAs production and recovered integrity of gut barrier in PD mice.

### OFO deactivates microglia and reduces neuroinflammation

**Figure 3:**
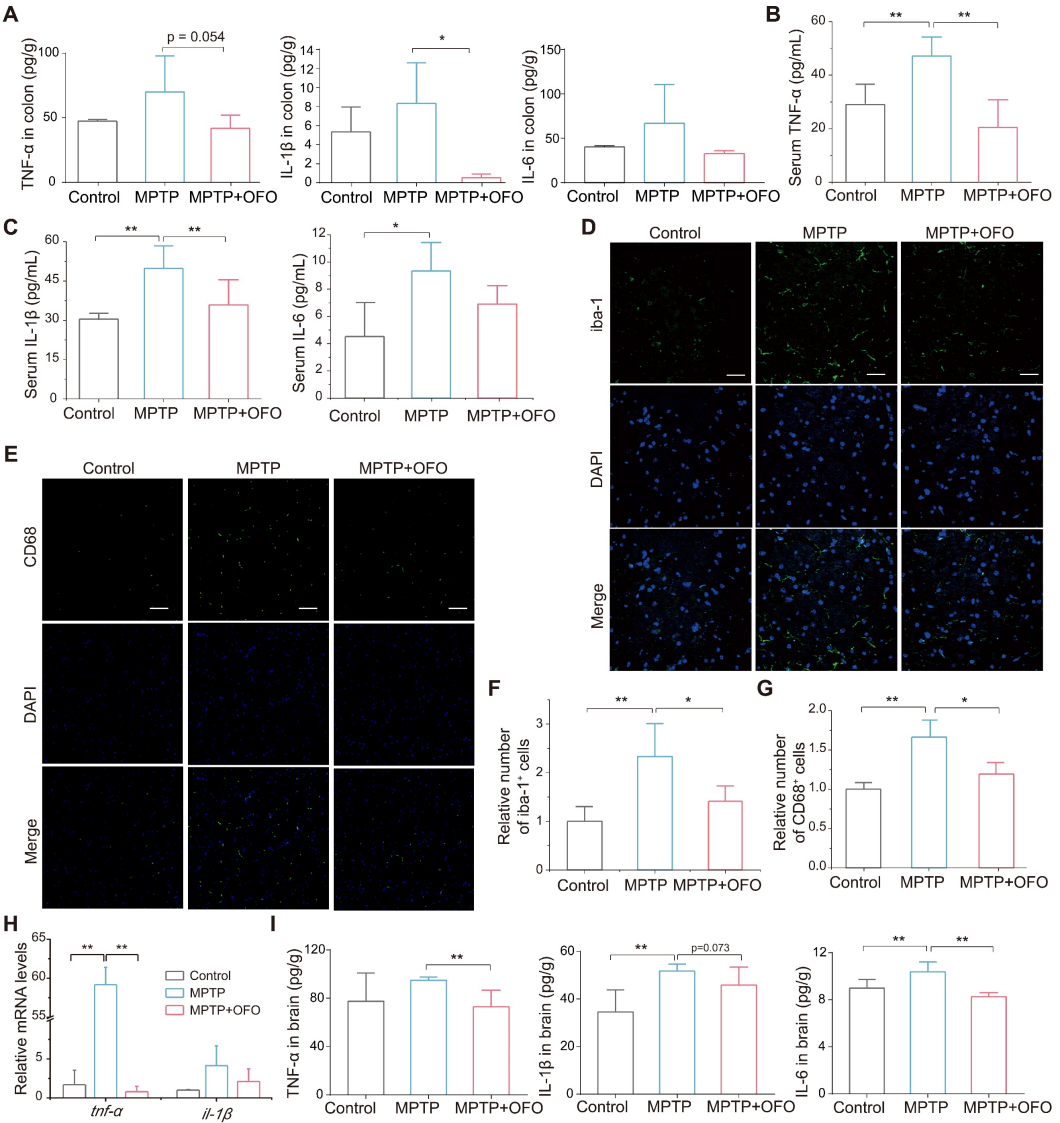
** OFO deactivated microglia and reduced neuroinflammation.** (**A**) The contents of TNF-α, IL-1β, and IL-6 in colon tissues. (**B-C**) The levels of TNF-α, IL-1β, and IL-6 in the serum. (**D**) Fluorescence microscopy of iba-1+ (green) microglia and DAPI (blue) in substantia nigra. Scale bars represent 40 μm. (**E**) Fluorescence microscopy of CD68 (green) and DAPI (blue) in substantia nigra. Scale bars represent 60 μm. (**F**) Quantitative analysis of immunofluorescence of iba-1. (**G**) Quantitative analysis of immunofluorescence of CD68. (**H**) The mRNA expression of* tnf-α* and *il-1β* in the midbrain. (**I**) The contents of TNF-α, IL-1β, and IL-6 in brain tissues. Data are the mean ± S.D. n = 3-6 in each group. *p < 0.05 and **p < 0.01. Data were analyzed using one-way ANOVA with LSD test or Games-Howell test by SPSS.

Notably, the intestinal and systemic inflammation was evaluated via testing the main pro-inflammatory cytokines such as tumor necrosis factor-α (TNF-α), interleukin-1β (IL-1β), and IL-6 in both colonic tissues and serum. Our results revealed that the levels of these cytokines in colon were increased in PD mice, but decreased in OFO-treated mice (**Figure 3A**). Importantly, OFO also reduced TNF-α, IL-1β, and IL-6 levels in serum (**Figure 3B-C**), indicating that OFO could alleviate both local and systemic inflammation. Furthermore, microglia are a kind of brain-resident macrophages, whose activation was evaluated by the immunofluorescence staining of ionized calcium binding adapter molecule-1 (iba-1) and CD68 24, 25. It manifested that OFO significantly decreased the numbers of iba-1 and CD68 positive cells in substantia nigra (**Figure 3D-G**), suggesting the deactivation of microglia by OFO after lowering systemic inflammation. In particular, OFO also decreased the mRNA expressions of *tnf-α* and *il-1β* in midbrain (**Figure 3H**) and lowered the protein levels of TNF-α, IL-1β, and IL-6 in brain (**Figure 3I**). Collectively, the impairment of microglia activation and decrease of pro-inflammatory cytokines levels by OFO both indicated that OFO reduced neuroinflammation in MPTP-induced PD mice.

### OFO improves motor deficits in MPTP-induced PD mice

**Figure 4:**
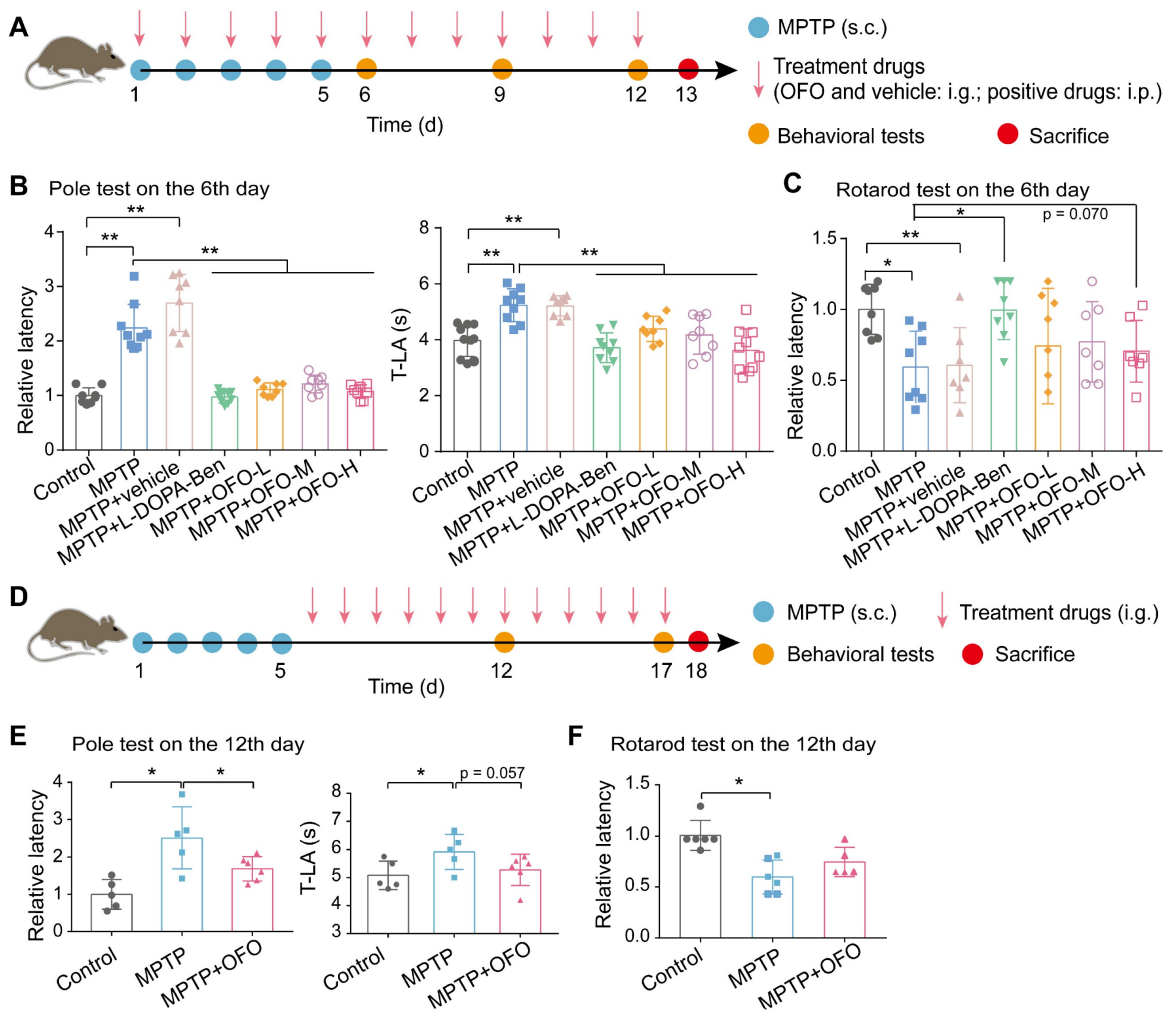
** OFO improves motor deficits of MPTP-induced PD mice.** (**A**) Schematic diagram depicted the experimental protocol of the prevention against MPTP-induced PD by OFO. (**B-C**) The pole test (B) and the rotarod test (C) on the 6th day in the prevention experiments. (**D**) Schematic diagram depicted the experimental protocol of the treatment of MPTP-induced PD by OFO. (**E-F**) The improvement of MPTP-induced behavioral dysfunction by OFO, as assessed by the pole test (E) and the rotarod test (F) on the 12th day. Data are the mean ± S.D. n = 3-10 in each group. *p < 0.05 and **p < 0.01. Data were analyzed using one-way ANOVA with LSD test or Games-Howell test by SPSS.

Based on the anti-neuroinflammation functions of OFO, we evaluated the therapeutic effects of it on PD. First, MPTP-induced PD mouse model was constructed. The MPTP (s.c.) combined with OFO (i.g.) was sequentially administrated for the consecutive 5 days, and we terminated MPTP but continued to administrate OFO for extra 7 days with levodopa (L-DOPA) and benserazide (i.p.) as positive drugs (**Figure 4A**). The behavioral tests including pole test and rotarod test were performed on the 6th, 9th and 12th day.

The latency and the time to reach the base of the pole (T-LA) in pole test were both significantly prolonged in MPTP-induced PD mice, but were decreased by positive drugs and OFO in a concentration dependent manner (**Figure 4B** and **Figure S5A-B**). The latency in rotarod test was also improved both by positive drugs and OFO (**Figure 4C** and **Figure S5C-D**). Moreover, no significant reductions of these values were observed in PD mice treated by the vehicle of olive oil alone. To further explore the treatment effects of OFO on PD, another group of PD mice was set up by OFO for 12 days after 5-day MPTP injection, and the behavior tests were performed on the 12th and 17th day (**Figure 4D**). Consistently, the latency and T-LA in pole test were also reduced by OFO (**Figure 4E** and **Figure S6A**). And the latency in rotarod test exhibited the tendency to increase after OFO treatment (**Figure 4F** and **Figure S6B**). Altogether, these results demonstrated that OFO ameliorated the bradykinesia and motor incoordination of PD mice induced by MPTP.

### OFO reverses DA loss in MPTP-induced PD mice

**Figure 5:**
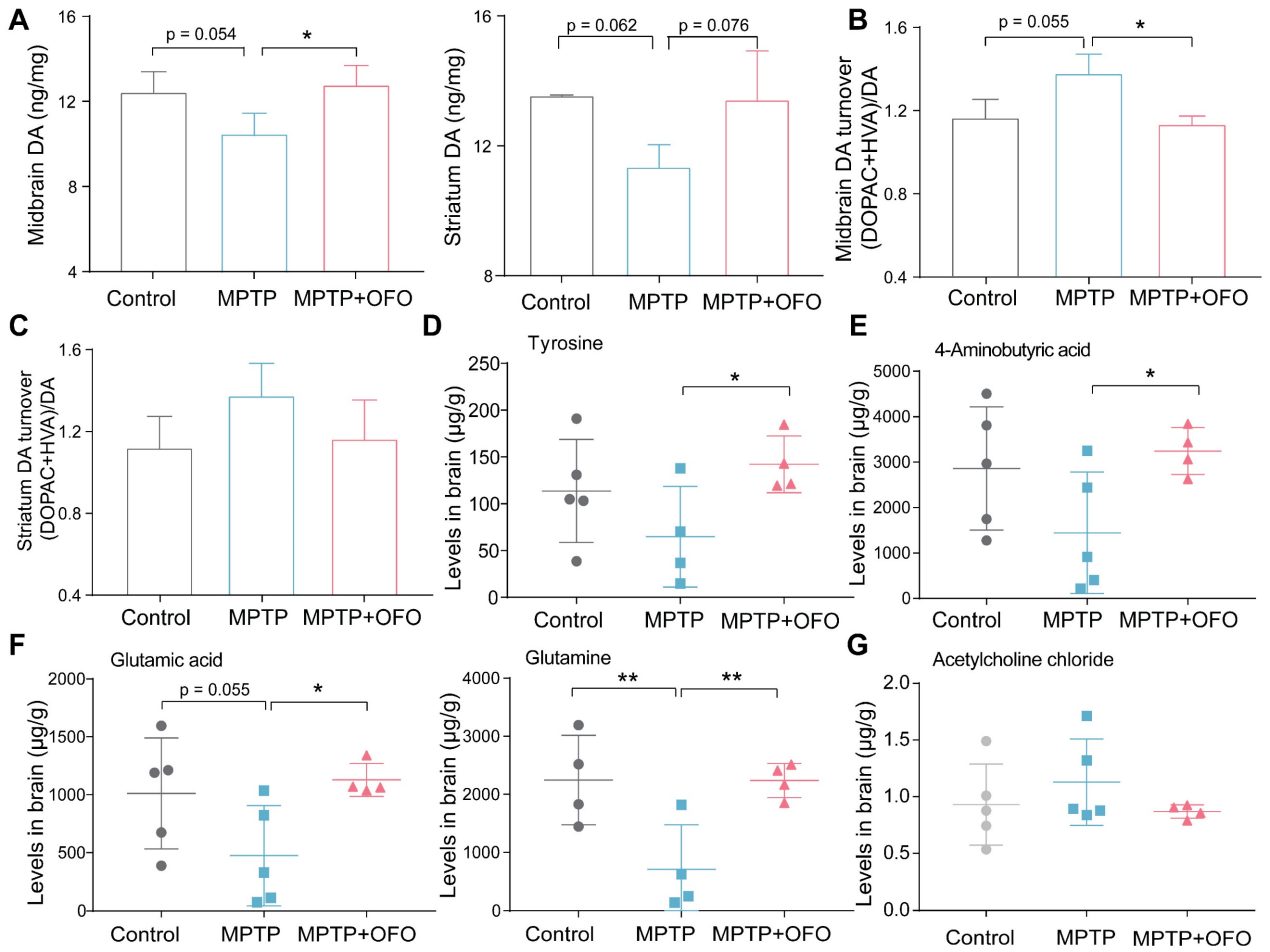
** The effects of OFO on neurotransmissions of MPTP-induced PD mice.** (**A**) The contents of DA in the midbrain and striatum. (**B-C**) DA turnover ratio (DOPAC+HVA)/DA in the midbrain (B) and striatum (C). (**D**) The levels of tyrosine in brain tissues. (**E-F**) The levels of 4-aminobutyric acid, glutamic acid, and glutamine in brain tissues. (**G**) The levels of acetylcholine chloride (Ach) in brain tissues. Data are the mean ± S.D. n = 4-6 in each group. *p < 0.05 and **p < 0.01. Data were analyzed using one-way ANOVA with LSD test or Games-Howell test by SPSS.

It is recognized that DA loss is the most typical neuropathological manifestations of PD 26. Thus, we evaluated the effects of OFO on MPTP-induced DA loss. The contents of DA in the midbrain and striatum were decreased after MPTP administration. Excitingly, OFO highly reversed MPTP-induced DA deficiency (**Figure 5A**). 3,4-Dihydroxyphenylacetic acid (DOPAC) and homovanillic acid (HVA) were two kinds of metabolites of DA. Our results indicated that the turnover ratio of DA ((DOPAC+ HVA)/DA) in MPTP treated mice was markedly increased. Intriguingly, the DA turnover ratio was obviously decreased after OFO treatment, suggesting that OFO could reduce DA catabolism (**Figure 5B-C**). Importantly, tyrosine, a DA precursor, was remarkably enlarged by OFO (**Figure 5D**). These results revealed that OFO alleviated DA loss for PD treatment. Converging evidence supports that disturbances in neurotransmitter systems beyond dopamine system exist in PD and impact dopaminergic circuits 27, 28. The relevant non-dopaminergic neurotransmissions in brain were therefore evaluated in our work, such as 4-amino butyric acid (GABA), glutamatic acid (Glu), glutamine (Gln), and acetylcholine chloride (Ach). We found that OFO significantly elevated GABA, Glu, and Gln levels in brain tissues (**Figure 5E-F**), and OFO tended to reduce the level of Ach (**Figure 5G**), which antagonizes DA action. It revealed that OFO could reverse the disturbances of these non-dopaminergic neurotransmissions for PD treatment.

### OFO prevents MPTP-induced dopaminergic neuronal degeneration

**Figure 6:**
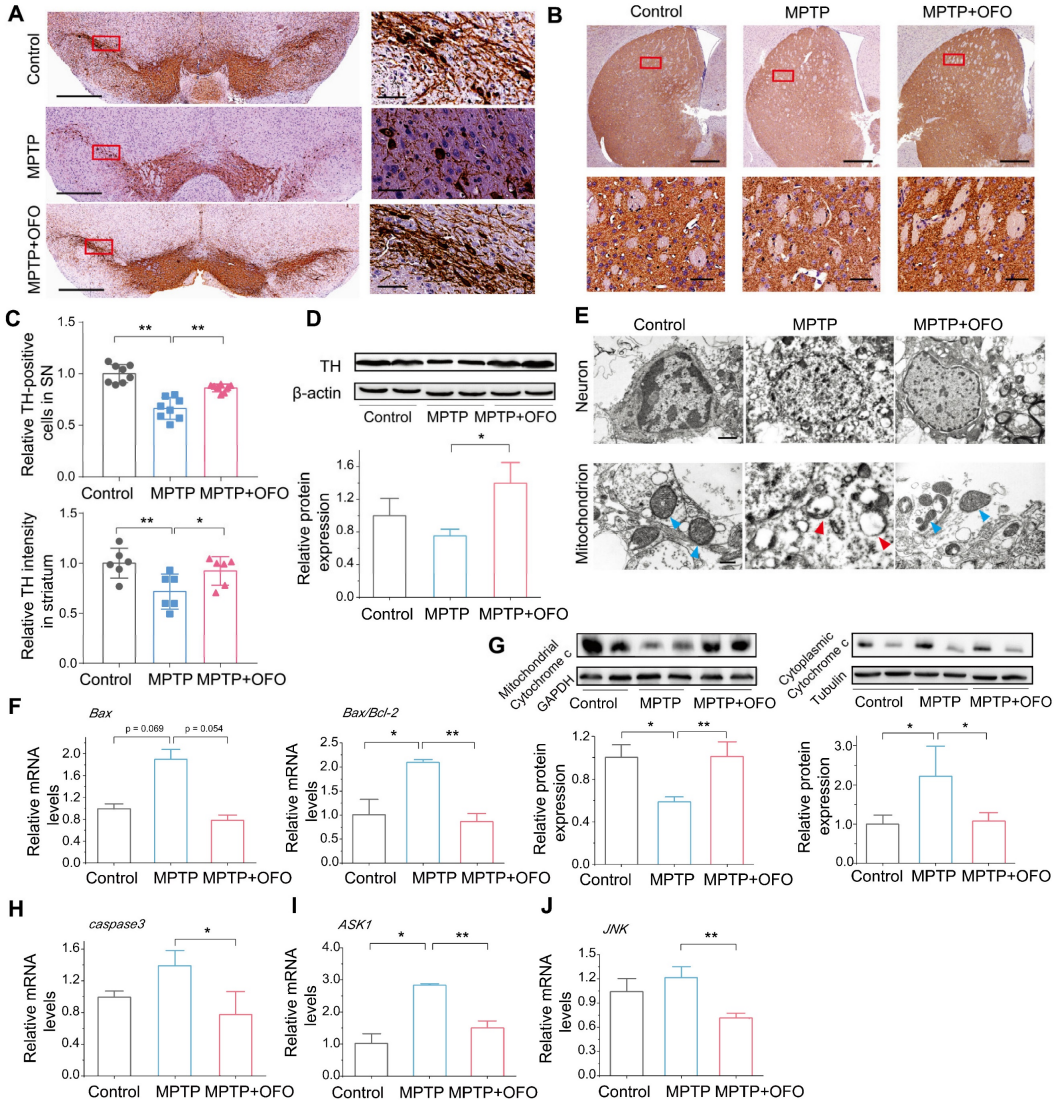
**The prevention of dopaminergic neuronal degeneration by OFO in MPTP-induced PD mice.** (**A**) IHC images of TH in SN. The right pictures are the magnification of the red rectangle in the left. The bar: left 500 μm, right 50 μm. (**B**) IHC images of TH in striatum. The bottom pictures are the magnification of the red rectangle in the top. The bar: top 500 μm, bottom 50 μm. (**C**) Quantitative analysis of TH-positive neurons in SN and in striatum. (**D**) WB and quantitative analysis of TH in the midbrain. (**E**) The ultrastructure of dopaminergic neurons and mitochondrion in the control, MPTP, and MPTP+OFO groups by TEM. Scale bar: 1 μm and 400 nm. The blue arrows refer to the normal mitochondria. The red arrows refer to the damaged mitochondria. (**F**) The mRNA expression of *Bax* and the ratios of *Bax/Bcl-2* in the midbrain by Q-PCR analysis. (**G**) The protein expression of cytochrome c in the mitochondrion and cytoplasm of neurons in the midbrain by WB. (**H**) The mRNA expression of *caspase3* in the midbrain. (**I-J**) The mRNA expression of *ASK1* (I) and* JNK* (J) in the midbrain. Data are the mean ± S.D. n = 3-6 in each group. *p < 0.05 and **p < 0.01. Data were analyzed using one-way ANOVA with LSD test or Games-Howell test by SPSS.

To investigate the effects of OFO on dopaminergic neuronal degeneration in PD mice, we quantified the tyrosine hydroxylase (TH) expressing dopaminergic cells in substantia nigra (SN) and striatum by IHC. Obviously, MPTP reduced surviving TH expressing neurons in number. However, there was a remarkable recovery of TH expressing neurons after OFO treatment (**Figure 6A-C**). Additionally, the promotion of TH protein expressions in the midbrain of OFO treated mice also consisted with the above-mentioned results (**Figure 6D**). Then, we observed the ultrastructure of dopaminergic neurons in SN using TEM. It delineated that the dopaminergic neurons in PD mice were seriously damaged with fractured cell structure, discontinuous nuclear membrane, disappeared nucleoli and pyknotic chromatin, and the mitochondria badly suffered disruption with vacuolization and disintegrated cristae after MPTP injection (**Figure 6E**). Interestingly, OFO impaired these injuries. However, the positive drugs could not improve the neuronal and mitochondrial structure, indicating that it could not reduce the neuronal degeneration by exogenous supplements of DA during the observation period (**Figure S7**). In addition, we found that MPTP induced mitochondria-mediated neuronal apoptosis with the increase of mRNA levels of *Bcl2-associated X (Bax)* and *Bax/B-cell lymphoma-2 (Bcl-2),* the release of cytochrome c to cytoplasm from mitochondria, and the promotion of *caspase3* mRNA expression (**Figure 6F-H**). Intriguingly, OFO reversed these abnormities, suggesting the suppression of mitochondria-mediated neuronal apoptosis after OFO treatment. Furthermore, we also demonstrated that OFO observably down-regulated the mRNA levels of *apoptosis signal-regulating kinase 1* (*ASK1*) and *c-Jun N-terminal kinase* (*JNK*) (**Figure 6I-J**), showing that OFO also inhibited neuronal apoptosis via ASK1/JNK signal pathway. All these results indicated that OFO treatment prevented dopaminergic neuronal degeneration.

### OFO exhibits low toxicity in vivo

**Figure 7:**
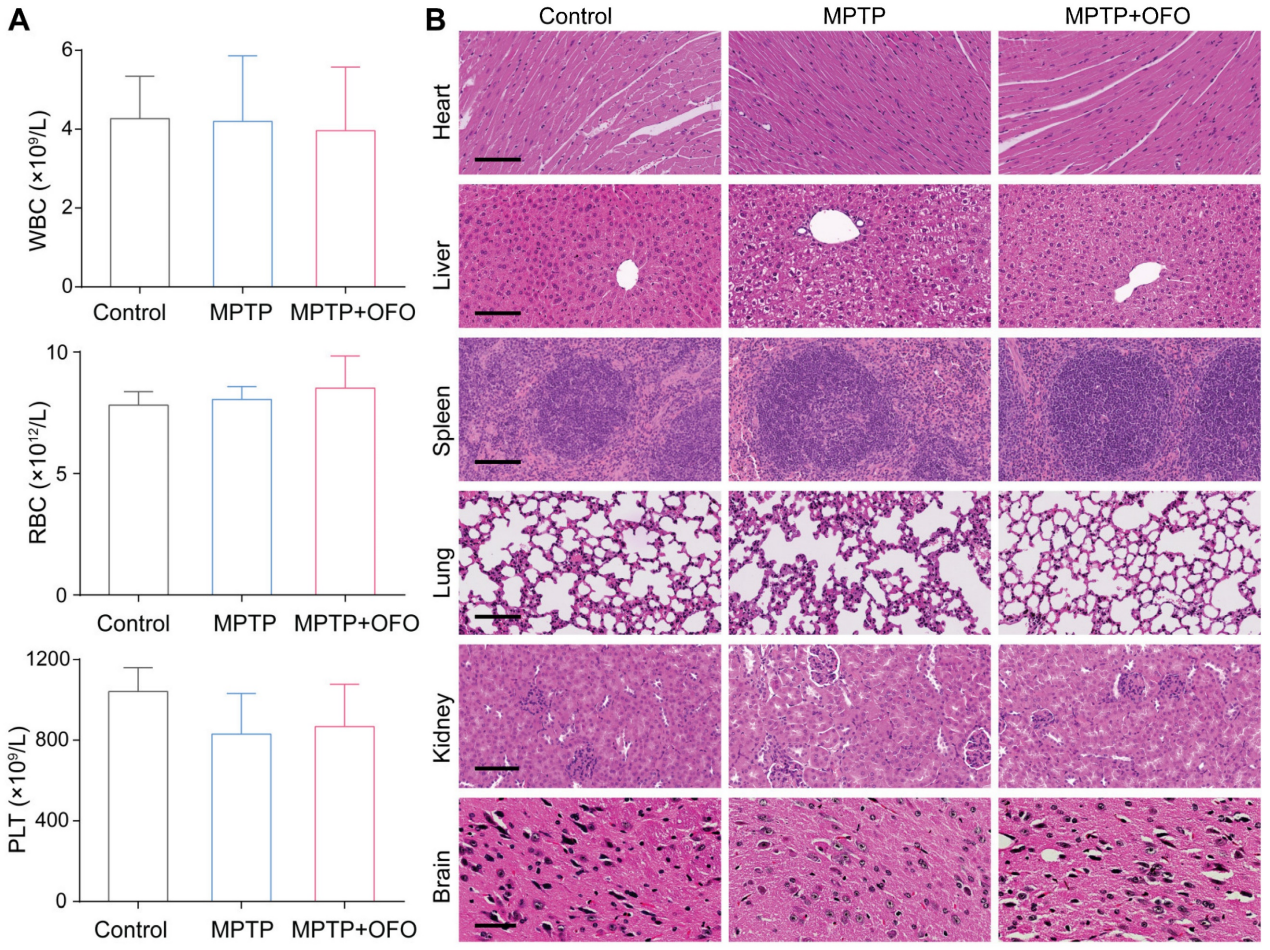
** Toxicity evaluation.** (**A**) Routine blood test for WBC, RBC and PLT of mice in different groups. (**B**) HE staining of heart, liver, spleen, lung, kidney and brain in different groups. Scale bars of heart, liver, spleen, lung and kidneys represent 100 μm, and scale bar of brain represents 50 μm.

Moreover, the toxicity of OFO was evaluated by mouse weight, blood test, and histopathological analysis in PD mice. During the 12-day prevention of PD mice, we measured the body weights of mice everyday, founding that no significant weight decreases were observed after OFO treatment compared with MPTP groups (**Figure S8**). The contents of white blood cells (WBC), Red blood cells (RBC), and platelet (PLT) exhibited normal levels after treated by OFO compared with the control mice (**Figure 7A**), suggesting no detectable damage of OFO to blood cells. The main organs such as heart, liver, spleen, lung, kidneys and brain were stained with hematoxylin and eosin (HE), and no obvious inflammation, cellular necrosis and apoptosis appeared. In addition, we surprisingly found that OFO prominently protected against MPTP-induced hepatic damage and dopaminergic neuronal damage in SN of brain tissues (**Figure 7B**). These suggested the low toxicity of OFO towards the tested mice.

## Discussion

Neuroinflammation appears as a central event in neurodegeneration pathophysiology, providing a promising therapeutic strategy for PD 29, 30. To date, it has attracted considerable attentions for anti-inflammatory strategies to treat neurodegenerative disorders via regulating microglia, inhibiting the cyclooxygenase (COX) activity, or targeting cytokine signaling 31, 32. However, the current clinical anti-inflammation drugs for treating neurodegeneration are unsatisfactory with limitations of the shielding effects of BBB, the unpredictable side-effects, the feasibility of long-term administration 33, 34. Excitingly, we presented a promising therapeutic medication of oral [60]fullerene for PD through suppressing neuroinflammation via regulating the diversity of gut microbiome, increasing the contents of SCFAs, and recovering the integrity of gut barrier.

Extensive studies supported that gut microbiota could regulate neuroinflammation 35. Particularly, the dysbiosis of gut microbiota and its metabolites jeopardized gut structure and aggravated intestinal inflammation, releasing pro-inflammatory factors and increasing systemic inflammation 36. Generally, the circulative pro-inflammatory cytokines could enter into brain tissues and activate microglial cells, inducing brain inflammatory response. The activated microglial cells would release cytotoxic inflammatory compounds, such as pro-inflammatory cytokines, to exaggerate neuroinflammation and lead to ultimate neuronal degeneration 37, 38. So, gut microbiota intervention could be a potential method to regulate neuroinflammation and neurodegenerative diseases. Actually, it has been explored to intervene neurodegenerative diseases by gut microbiota, such as probiotics treatment, fecal microbiota transplantation, and other bioactive substances uptake 39-42. However, there are increasing limitations in these approaches, like the instability of the bacteria and bioactive substances in gastric fluid, low efficiency and bioavailability, microbiota disorders, complicated operation, poor patient acceptance and so forth 43. Excitingly, our study reported a superior gut microbiota regulator of OFO, which distributed in gastrointestinal tract contents, then increased the beneficial bacteria (*Bacteroides, Akkermansia, Faecalibaculum, and Parabacteroides*), enriched acetic acid and butyric acid, and elevated tight junction proteins expression. These regulations reduced colonic and systemic inflammation, and eventually dampening neuroinflammation.

The current anti-PD medications such as levodopa, dopamine agonist, monoamine oxidase B inhibitors, amantadine, and catechol-O-methyltransferase inhibitors usually possess some defects with low bioavailability (difficult to cross BBB), dopaminergic motor complications and wearing off 44-46. Thus, breakthroughs still need to achieve in anti-neurodegeneration drug development. Here, we demonstrated that OFO exerted excellent therapeutic effects on PD with improvement of motor deficits and DA loss. Importantly, OFO alleviated neuronal degeneration, instead of relieving symptoms. Actually, accumulating studies reported that water-soluble fullerene derivatives could prevent oxidative stress, mitochondrial dysfunction, and inflammation, exerting neuroprotective properties 47-63. However, these fullerenes were all injected by intravenous injection, intraperitoneal injection, intracerebroventricular infusion or osmotic pump uptake. It is rarely reported that fullerenes treated PD via indirectly regulating gut microbiota, but not via directly reacting in brain.

The therapeutics for PD by OFO showed evident advantages. (1) OFO was stable and difficult to be destroyed by acid and enzymes, resulting in the possibility of oral administration, which could be more acceptable in clinic. (2) OFO reduced neuroinflammation via regulating gut microbiome with no need for crossing BBB, which is a major limitation for clinical anti-PD drugs. (3) OFO prevented dopaminergic neuronal degeneration and increased endogenous DA contents, instead of exogenous DA supplement and symptoms relief, which are the primary strategies of the present clinical medications. (4) Almost all OFO was excreted through the gastrointestinal tract, exhibiting high biocompatibility and biosafety.

Altogether, OFO distributed in gastrointestinal tract contents, regulated gut microbiome with enhanced beneficial bacteria, increased SCFAs production, and integral gut barrier, resulting in reducing colonic and systemic inflammation, and thereby inhibiting microglia activation and pro-inflammatory cytokines releases. Thus, this reduction of neuroinflammation prevented dopaminergic neurodegeneration, which improved motor dysfunction and reversed DA loss of PD mice. We proposed an anti-neurodegeneration strategy based on anti-inflammatory OFO, providing pathological basis and therapeutic candidates for neuroinflammation-related neurodegenerative diseases.

## Materials and Methods

### Chemical and reagents

C_60_ fullerene was obtained from Beijing Fullcan Co. Ltd., China. Oliver oil was purchased from Coreysa (Spain). 1-methyl-4-phenyl-1,2,3,6-tetrahydropyridine hydrochloride (MPTP), levodopa (L-DOPA) and benserazide (Ben) were purchased from Sigma-Aldrich (USA). Donepezil was obtained from Adamas (China). The anesthetic of avertin made up by 2,2,2-tribromoethanol and 2-methyl-2-butanol and calcium colorimetric assay kit were purchased from Sigma-Aldrich. Elisa kits of dopamine (DA), 3,4-dihydroxyphenylacetic acid (DOPAC), homovanillic acid (HVA), interleukin-1β (IL-1β), tumor necrosis factor-α (TNF-α), and interleukin-6 (IL-6) were purchased from Shanghai enzyme-linked Biotechnology Co. Ltd. (China). The primary antibody of tyrosine hydroxylase (TH), occludin, claudin1, Muc2, CD68, and cytochrome c were purchased from Abcam (UK). The mitochondria/cytosol fractionation kits were purchased from Abcam (UK). All reagents and solvents were commercially obtained and used without further purification.

### Preparation and characterization of OFO

OFO was prepared according to the previously reported reference 64. Briefly, 100 mg of C_60_ were dissolved in 100 mL of olive oil, and then the mixture was stirred at ambient temperature in the dark using a ball mill (QM-QX2L, MITR, 600 rpm/min, 30 min, agate grinding beads with diameter of 10 mm). After centrifuged at 5,000 r/min for 1 h, the resulting supernatant was filtered through Millipore filter with 0.22 μm porosity.

The purity of the C_60_ was characterized by high performance liquid chromatography (HPLC, LC-2030, Shimadzu, Japan) on a buckyprep column (chromatographic column 4.6 × 250 mm, flow rate 1 mL/min, toluene as eluent, wavelength 310 nm) and matrix assisted laser desorption ionization time of flight mass spectrometry (MALDI-TOF MS, AXIMA Assurance, Shimadzu, Japan).

OFO was mixed with anhydrous acetic acid and hydrochloric acid (v/v, 1/2), and the system was heated to 80 °C for 30 min. And then, the sample was extracted by toluene for MALDI-TOF MS and for HPLC to determine the concentration of C_60_ in OFO solution. OFO at the concentration of 6.25 μg/mL was detected by ultraviolet-visible (UV-vis) spectra (UH4150, Hitachi, Japan). To examine the ROS-scavenging ability, OFO was first emulsified by adding Tween 20 (v/v, 1/3) and ultrapure water (v/v, 1/6). Then, the solution was mixed with 5,5-dimethyl-1-pyrroline-noxide (DMPO) and H_2_O_2_ with concentration of 12.5 μg/mL and detected by electron paramagnetic resonance spectrometer (EPR, E500, Bruker, Germany).

### Animals

Male C57BL/6J mice (18-20 g, 6-8 weeks old) were purchased from Sippr-B&K Laboratory animal Co. Ltd. (China). Mice were housed under a 12 h light/dark cycle in a temperature-controlled, ventilated and standardized animal room and fed with food and water *ad libitum*. All the experimental protocols involving live animals were approved by the Animal Ethics Committee of Institute of Chemistry, Chinese Academy of Sciences (CAS).

### Biodistribution studies

Male C57BL/6J mice were treated orally by OFO at the dosage of 6.5 mg/kg (n = 3). After treatment for 1, 4, 8, 24 h and 7 d, groups of mice were sacrificed for blood and organ collection (heart, liver, spleen, lung, kidneys, brain, stomach and intestinal tract). For the whole blood, 50 μL of sample were added to 50 μL o-xylene and mixed. For organs, samples were weighted and homogenized with deionized water (w/v, 1:4). After adding anhydrous acetic acid and hydrochloric acid (v/v, 1/2), the samples were heated to 80 °C for 30 min and extracted by o-xylene. After mixed with acetonitrile, the samples were analyzed by liquid chromatography-mass spectrometry (LC-MS, API5500, SCIEX, USA). The contents of samples were quantified by internal standard method. The whole blood and organs of mice without OFO treatment spiked with OFO solutions were used for validating the method.

### 16S rRNA gene sequencing analysis of gut microbiota

After 12 days drug administration, feces were collected using metabolic cages and then frozen in -80 °C. Total gene was extracted and quantified with a Qubit 2.0 Fluorometer (Invitrogen, USA). 30 - 50 ng DNA and V3 and V4 regions of 16S rRNA were selected for generating amplicons. The microbiol genes were amplified using forward primers of the sequence of “CCTACGGRRBGCASCAKVRVGAAT” and reverse primers of the sequence of “GGACTACNVGGGTWTCTAATCC”. After purification, DNA libraries were loaded on an Illumina MiSeq instrument under the manufacturer's instructions (Illumina, USA). Sequencing was performed using a 2 x 300 paired-end (PE) configuration. Data analyses were conducted using the QIIME (Quantitative Insights Into Microbial Ecology) software package by a specialist.

### SCFAs detection

SCFAs were evaluated by the gas chromatography mass spectrometer (GC-MS, TRACE 1310-ISQ LT, Thermo, USA). Briefly, 50 mg of feces samples were suspended in 50 μL of 15% phosphoric acid and 4 μL ether with 125 μg/mL of isocaproic acid as internal standard. Then, the solution was strongly vortexed for 1 min. After centrifuged at 12000 rpm at 4 °C, the supernatant was collected and examined by GC-MS equipped with Agilent HP-INNOWAX column (30 m * 0.25 mm * 0.25 μm). The program was performed as follows: started at 90 °C, heated to 120 °C by 10 °C/min, heated to 150 °C by 5 °C/min, heated to 250 °C by 25 °C/min and held for 2 min. Then, the SCFAs contents were analyzed.

### Quantitative real-time PCR (Q-PCR)

Briefly, total RNA was extracted from tissues of treated and untreated animals by the trizol isolation reagent according to the manufacturer's instructions. After some precipitation, separation and purification, the RNAs were reverse- transcribed into cDNA with revert aid first strand cDNA synthesis kit (K1622, Fermentas, Lithuania). The primers for PCR were listed in Table S1. According to the manufacturer's guidelines, real-time PCR was performed with the 2×SYBR green mastermix (Q111-01, Vazyme, China). The results were calculated via the 2^-ΔΔCt^ method using the threshold cycle (Ct) value, and reported as fold change in gene expression.

### Western blot (WB) analysis

Following sacrifice, each animal's brain and colons was rapidly removed and the ventral midbrain containing the SNpc was dissected out. Isolated tissues were rinsed with cold PBS and homogenized in lysis buffer containing protease inhibitor cocktail for 1 hour. And then lysates were centrifuged at 13000 rpm at 4 °C for 3-5 minutes. The total protein contents in the supernatant were determined by a bicinchoninic acid (BCA) protein assay kit (Cwbiotech, China). The protein samples in the cytoplasm and mitochondria were extracted according to the mitochondria/cytosol fractionation kits. The samples were separated by sodium dodecyl sulfate polyacrylamide gel electrophoresis (SDS-PAGE) on 10% polyacrylamide gels and electro-transferred to polyvinylidene difluoride (PVDF) membranes. The membranes were incubated with a blocking buffer for 1 hour at room temperature, and then incubated with the primary antibody at 4 °C overnight. The next day, after rinsed three times with Tris buffered saline tween (TBST), the membranes were incubated with the secondary antibody for 1 hour at room temperature. Protein bands were visualized and quantified by software of Gel Image system ver.4.00.

### Biochemical analysis

Blood serum was collected. After decapitation of animals, the brain was removed and tissues from the midbrain region were dissected out, frozen in liquid nitrogen and stored at -80 °C until assayed. And the mitochondrial solution was fracted according to the mitochondria/cytosol fractionation kits. DA and its metabolites 3,4-dihydroxyphenylacetic acid (DOPAC) and homovanilic acid (HVA) in midbrain tissues were examined using Elisa kits according to the introductions. The main factors including TNF-α, IL-1β, and IL-6 were examined according to the instructions of Elisa kits.

### Neurotransmitter analysis in brain

Brain tissues were grinded in 10% methanol-formate:ddH_2_O (1:1.v/v) solution. Then, after centrifuged at 12000 rpm at 4 ℃ for 5 min, the supernatant was collected and detected by LC-MS equipped with C18 column (2.1×100 mm, 1.7 μm, Waters), 10% methanol water (containing 0.1% formic acid) and 50% methanol water (containing 0.1% formic acid) as mobile phases. The MS is conducted using electrospray ionization (ESI) source in positive ion ionization mode.

### Histology and immunohistochemistry

After experiments (12 days' treatment in PD experiments), mice were sacrificed. And tissues were extracted and fixed with 4% paraformaldehyde, followed by dehydrate treatment and embedding in paraffin for histological, immunohistochemical, and immunofluorescence analysis. Following cutting and dewaxing, brain sections were initially washed in PBS for five minutes and then incubated in 3% methol-H_2_O_2_ for 15 minutes. After washing with PBS for three times, section were applied with high fire power in microwave oven for 4 minutes and low heat for 20 minutes and cooled to room temperature. Subsequently, they were incubated with primary antibodies at 4 °C overnight, then brain sections were subjected to secondary antibodies (Alexa Fluor® 488 conjugation for immunofluorescence) with 0.05% tween 20 for one hour at room temperature. After 3, 3-diaminobenzidine (DAB) coloration and hematoxylin counterstaining, the sections were counted after being hydrated again. All the pathological sections were scanned in their entirety with a NanoZoomer-SQ slide scanner (Hamamatsu, Japan). IHC analysis was conducted with the assistance of Image J software. The sections of immunofluorescence were observed by confocal microscopy (FV1000-IX81, Olympus, Japan).

### Animal experimental design in PD treatment experiment

The experiments were divided into two parts, one was the coadministration of OFO with MPTP, another was the administration of OFO after MPTP injection. For part one, the mice were distributed randomly into seven groups with ten replicates in each group: 1) the normal control group (Control, injected with saline (s.c.) and treated with saline (i.p.)), 2) the model control group (MPTP, injected with MPTP (s.c.) and treated with saline (i.p.)), 3) the olive oil group (MPTP+vehicle, injected with MPTP (s.c.) and treated with solvent olive oil (i.g.), 4) the positive drug group (MPTP+L-DOPA-Ben, injected with MPTP (s.c.) and treated with the positive drug L-DOPA and Ben (10 mg/kg and 2.5 mg/kg, i.p.)), the other three groups were OFO treatment groups with different concentrations (MPTP+OFO-L, MPTP+OFO-M and MPTP+OFO-H, injected with MPTP (s.c.) and treated with OFO (i.g.) at concentrations of 0.5655 mg/kg, 1.131 mg/kg and 2.262 mg/kg, respectively). Study comprised of total 12 days including five days of MPTP injection and twelve days drugs treatment. First, mice except in normal control group were subcutaneously injected with 25 mg/kg MPTP to induce moderate neurochemical and behavioural deficits, once daily for five consecutive days along with drugs treatment. The following seven days were just applied with different treatment drugs until the last day. The behaviour tests were conducted at 6 and 12 days to evaluate motor performance.

For another part, mice were distributed randomly into three groups with nine replicates in each group: 1) the normal control group (Control, injected with saline (s.c.) and treated with saline (i.g.)), 2) the model control group (MPTP, injected with MPTP (s.c.) and treated with saline (i.g.)), 3) the OFO treatment groups (MPTP+OFO, 1.131 mg/kg, i.g.). First, mice except in normal control group were subcutaneously injected with 25 mg/kg MPTP for 5 days, and then OFO was administrated for 12 days. The behaviour tests were conducted at 12 and 17 days to evaluate motor performance. The mice were terminally sacrificed and their blood, brain and intestinal contents were dissected for further investigations.

### Behavioral tests in PD experiment

The pole test was always used to measure bradykinesia in mouse PD models. The mice were held on the top of the pole (diameter 8 mm, height 55 cm, with a rough surface). The time that mice needed to turn down completely was recorded as the latency (time of turn). The time needed for the mice to climb down and place four feet on the floor was recorded as the T-LA (time for locomotion activity). Each trial had a cut-off limit of 30 s. Data were presented as the mean time over the five test trials.

The rotarod test was a method to measure motor coordination ability in mouse PD models. The rotarod unit consists of a rotating spindle and five individual compartments to test five mice at a time. For the test, mice were placed on the rod rotating at 28 rpm and the time each mouse remained on the rotating bar was recorded as latency for three trials, at a 5 min interval and a maximum trial length of 120 s per trial. Data were presented as the mean time on the rotating bar over the three test trials.

### Transmission electron microscopy (TEM)

After anesthetized by avertin, mice were intracardially perfused with saline solution followed by 2.5% glutaraldehyde and the brain was removed with tissues from the midbrain, striatum, hippocampus and cerebral cortex dissected. The tissues of approximately 1 mm^3^ were first fixed with a mixture of 2.5% (vol/vol) glutaraldehyde and 2% paraformaldehyde. And after washing with buffer solution of sodium dipotassium arsenate for three times, the tissues were fixed again using osmic acid. Then, after the tissues were dehydrated in an acetone gradient (30-100% for 5 min each), the samples were embedded in Epon 812 resin with different proportions of acetone and in 100% Epon 812 resin overnight at last. Ultrathin 70-mm sections were stained using uranyl acetate and lead citrate. Finally, the tumor sections were examined by TEM (Spirit 120 kV, FEI, USA).

### Statistics

All data reported were expressed as the mean ± standard deviation (S.D.). Statistical analyses of the samples were conducted using one-way ANOVA analysis with IBM SPSS Statistics 25 software.

## Supplementary Material

Supplementary figures and table.

## Abbreviations

PD: Parkinson's disease
MPTP: 1-methyl-4-phenyl-1,2,3,6-tetrahydropyridine
OFO: oral fullerene in olive oil
SCFAs: short chain fatty acids
DA: dopamine
BBB: blood brain barrier
MALDI-TOF MS: matrix-assisted laser desorption ionization time-of-flight mass spectrometry
UV-Vis: ultraviolet-visible
HPLC: high performance liquid chromatography
EPR: electron paramagnetic resonance
LC-MS: liquid chromatography-mass spectrometry
PCA: principal component analysis
PCoA: principal coordinates analysis
NMDS: nonmetric multidimensional scaling analysis
Q-PCR: quantitative polymerase chain reaction
WB: western blot
TNF-α: tumor necrosis factor-α
IL-1β: interleukin-1β
iba-1: ionized calcium binding adapter molecule-1
L-DOPA: levodopa
T-LA: the time to reach the base of the pole
DOPAC: 3,4-Dihydroxyphenylacetic acid
HVA: homovanillic acid
GABA: 4-amino butyric acid
Glu: glutamatic acid
Gln: glutamine
Ach: acetylcholine chloride
TH: tyrosine hydroxylase
SN: substantia nigra
IHC: immunohistochemistry
Bax: Bcl2-associated X
Bcl-2: Bax/B-cell lymphoma-2
ASK1: apoptosis signal-regulating kinase 1
JNK: c-Jun N-terminal kinase
WBC: white blood cells
RBC: Red blood cells
PLT: platelet
HE: hematoxylin and eosin
TEM: transmission electron microscopy
