# Supplementary material for: Oral [60]fullerene reduces neuroinflammation to alleviate Parkinson's disease via regulating gut microbiome
Source: Theranostics. 2023 Sep 4;13(14):4936–51. doi: 10.7150/thno.85711 (PMC10526674; doi:10.7150/thno.85711)
Supplement: Supplementary file 1 — Supplementary figures and table. [file thnov13p4936s1.pdf]

Supplementary data

Oral [60]fullerene reduces neuroinflammation to alleviate Parkinson's disease via regulating gut microbiome

Xue Li[1, 2], Ruijun Deng[1, 2], Jie Li[1,2], Hui Li[3], Zhe Xu[4], Lei Zhang[2,5], Linyin Feng[2,5], Chunying Shu[1,2], Mingming Zhen[1,2✉], Chunru Wang[1,2✉]

1. Beijing National Laboratory for Molecular Sciences, Key Laboratory of Molecular Nanostructure and Nanotechnology, CAS Research/Education Center for Excellence in Molecular Sciences, Institute of Chemistry, Chinese Academy of Sciences, Beijing 100190, China
2. University of Chinese Academy of Sciences, Beijing 100049, China
3. Beijing Fullcan Biotechnology Co. Ltd, Beijing, 100085, China
4. Chifeng Fullcan Biotechnology Co., Ltd, Inner Mongolia, 024099, China
5. Shanghai Institute of Materia Medica, Chinese Academy of Sciences, Shanghai 201203, China

✉Corresponding authors: M. Z. (zhenmm@iccas.ac.cn); C. W. (crwang@iccas.ac.cn)

Table S1. The primers for Q-PCR.

Primer	Primer sequence (5'-->3')	Products
**MouseACTB-F**	TTCTTGGGTATGGAATCCTGTG	203bp
**MouseACTB-R**	GGAGGAGCAATGATCTTGATCTT	
**JNK-f1**	TCCCCGATGTGCTTTTCC	
**JNK-r1**	GACGTTGATGTATGGGTGCTG	146bp
**ASK1-F1**	GTTAGGGAAGGGCACTTATGG	249bp
**ASK1-R1**	GAACGAAGGAGAGCAGAGAGG	
**Bax-F2**	CAGGATGCGTCCACCAAG	157bp
**Bax-R2**	TGCCATCAGCAAACATGTCA	
**Caspase-3-F1**	GGAACCCTTGTTTTGGACCT	181bp
**Caspase-3-R1**	CACACGGTTTTCCTCTCACATA	
**TNF-α-F1**	AAGAGGCACTCCCCCAAAA	127bp
**TNF-α-R1**	ATCACCCCGAAGTTCAGTAGAC	
**IL-1β-F2**	ATGATAACCTGCTGGTGTGTGA	257bp
**IL-1β-R2**	GGGTGTGCCGTCTTTCATTAC	
**Bcl-2-F3**	CTGGTTGGGATTCCTACGG	234bp
**Bcl-2-R3**	GGACGGCATGATCTTCTGTC	
**Occludin-F**	CAGTGTCTGTAAATCCCAGGATG	158bp
**Occludin-R**	ATGTACTGCCATGTCTGAAAGGT	
**Claudin1-F**	TATGACCCCTTGACCCCCAT	132bp
**Claudin1-R**	AGAGGTTGTTTTCCGGGGAC	

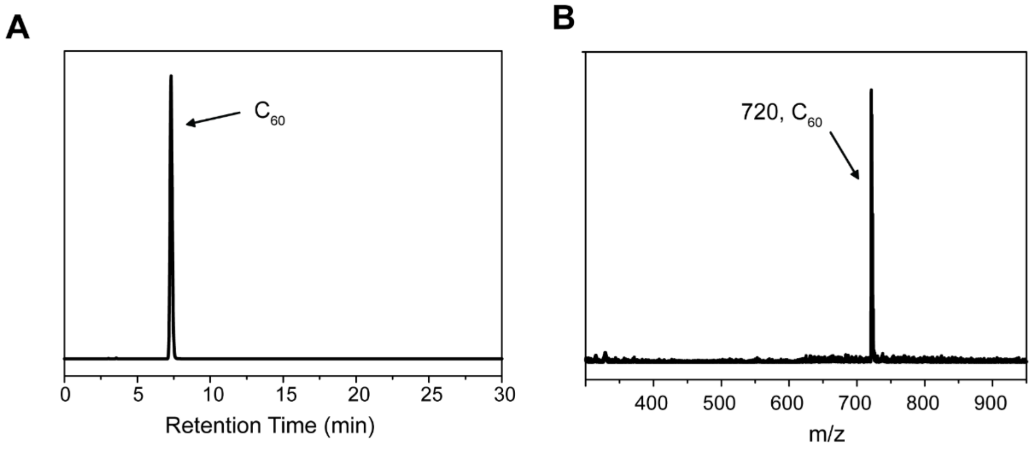

Figure S1. The characterization of C_{60}. (**A**) Chromatogram of the pristine C_{60} on a Buckyprep column (chromatographic column 4.6 × 250 mm; flow rate 1 mL/min; toluene as eluent; wavelength 310 nm). (**B**) MALDI-TOF-MS spectrum (positive mode) of the pristine C_{60}.

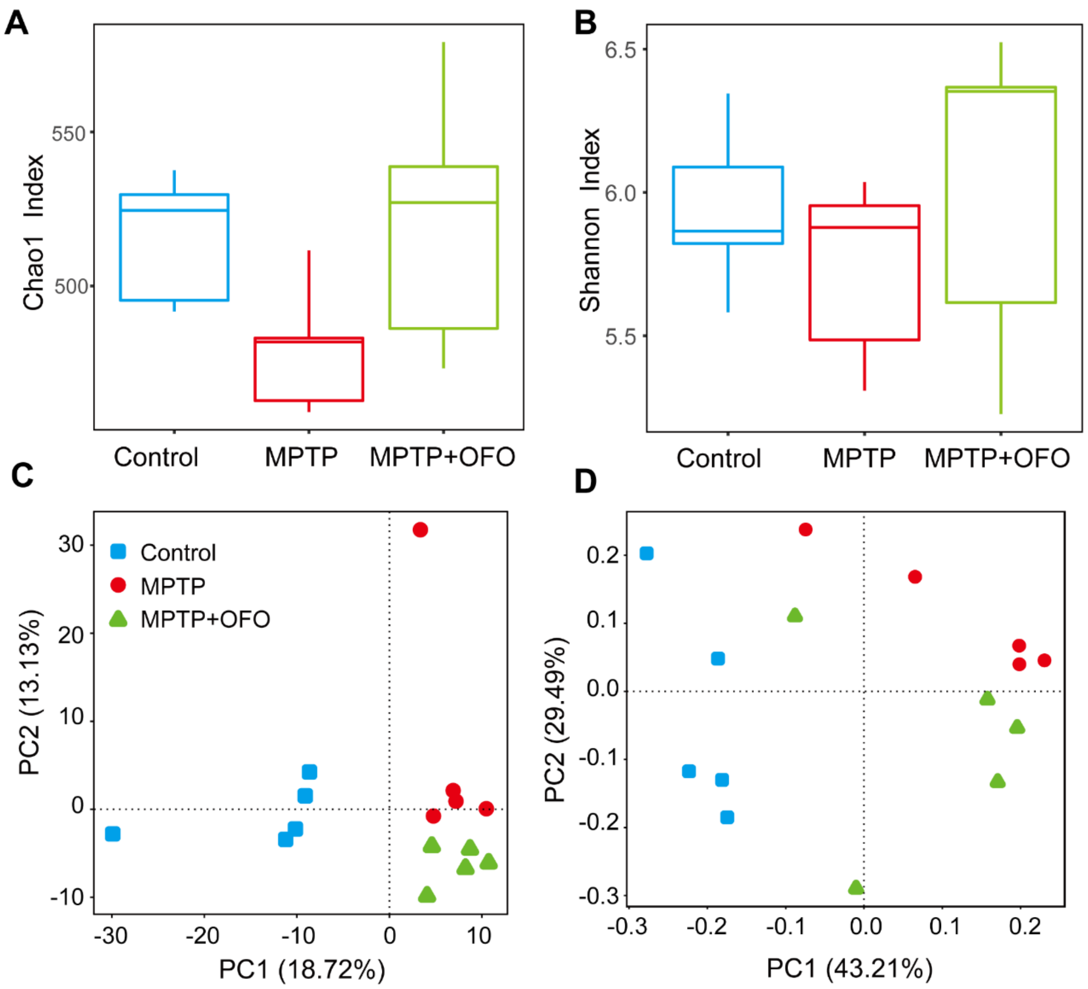

Figure S2. Effects of OFO on gut microbiota of MPTP-induced PD mice. (**A**) The Chao1 index. (**B**) The Shannon index (**C-D**) Analysis of the variances among the microbiol communities from the mice in the control, MPTP and treatment groups by the average relative abundance using principal component analysis (PCA, C) and principal coordinates analysis (PcoA, D).

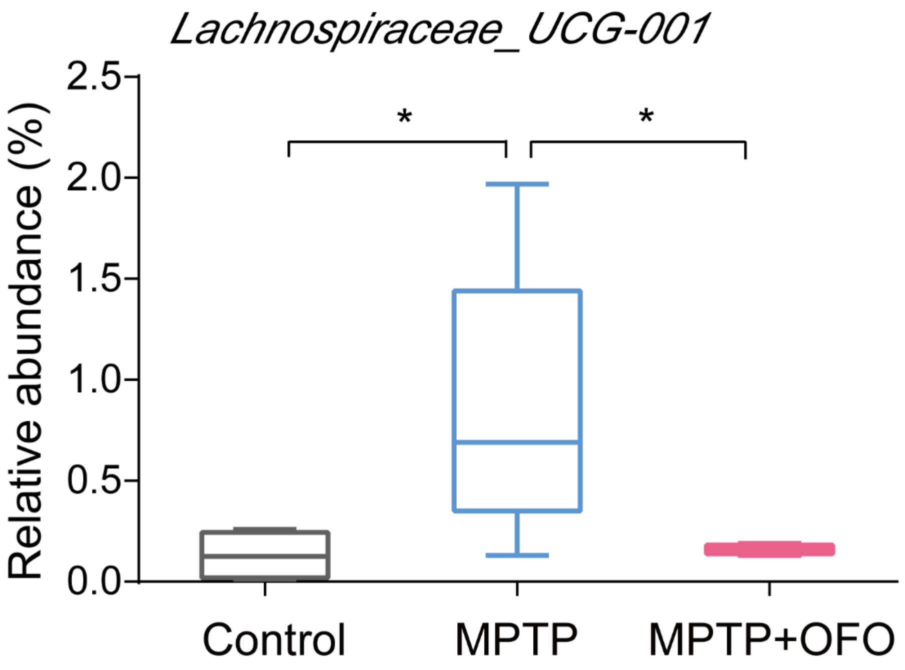

Figure S3. Relative abundance of *Lachnospiraceae_UCG-001* at genus level. *p < 0.05 and. Data were analyzed using one-way ANOVA with LSD test by SPSS.

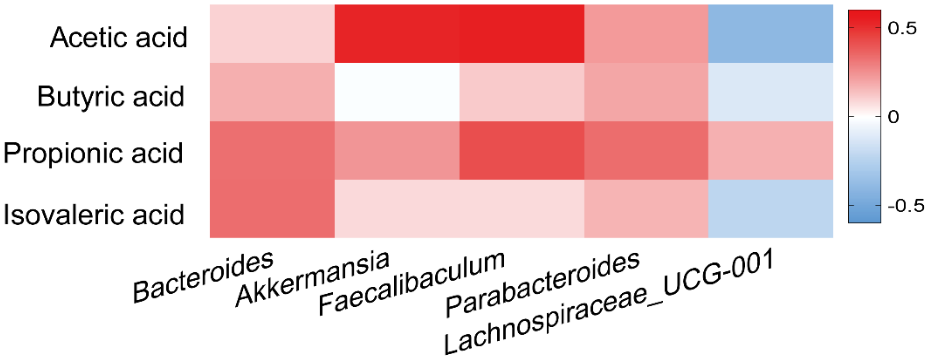

Figure S4. The heatmap of correlations between SCFAs and bacterial abundance at genus level.

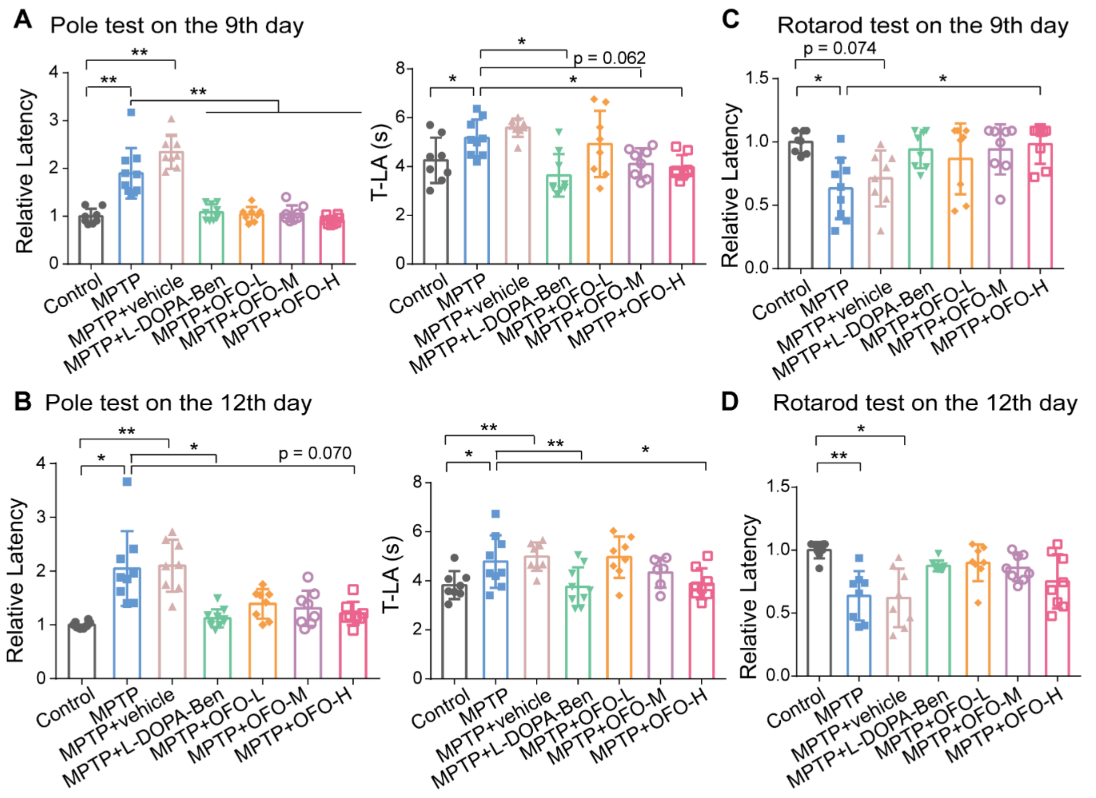

Figure S5. Effects of OFO on MPTP-induced motor deficits. The preventive effects of OFO on MPTP-induced behavioral injury, as assessed by the pole test and rotarod test on the 9th (A, C) and 12th days (B, D). Data are the mean ± S.D. n = 3-10 in each group. Data were analyzed using one-way ANOVA with LSD test or Games-Howell test by SPSS.

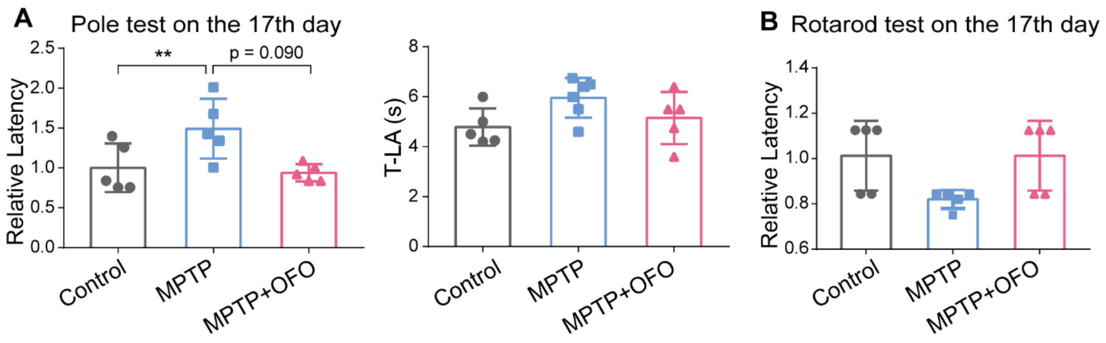

Figure S6. Effects of OFO on MPTP-induced motor deficits. The treatment of MPTP-induced behavioral injury by OFO, as assessed by the pole test (A) and rotarod test (B) on the 17th days. Data are the mean ± S.D. n = 6-10 in each group. Data were analyzed using one-way ANOVA with LSD test or Games-Howell test by SPSS.

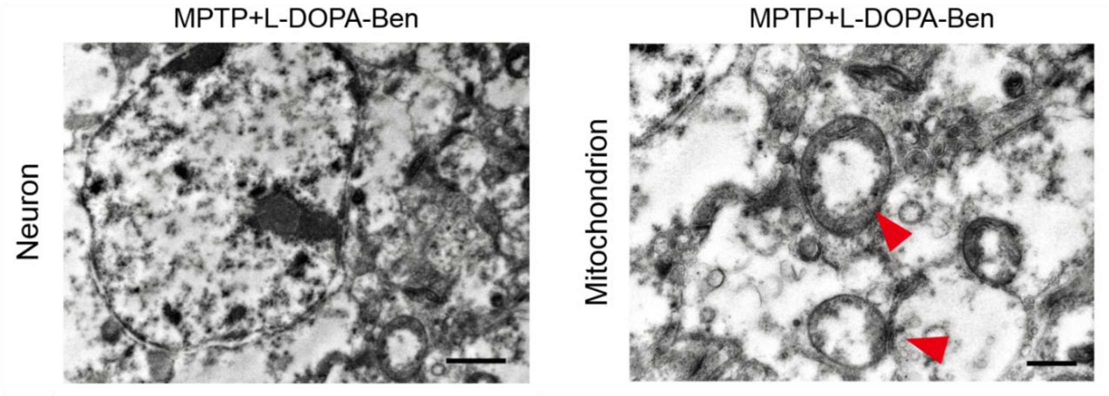

Figure S7. The ultrastructure of dopaminergic neurons and mitochondrion in the MPTP+L-DOPA-Ben group by TEM. Scale bar: 1 μm (left) and 400 nm (right). The red arrows refer to the mitochondrion.

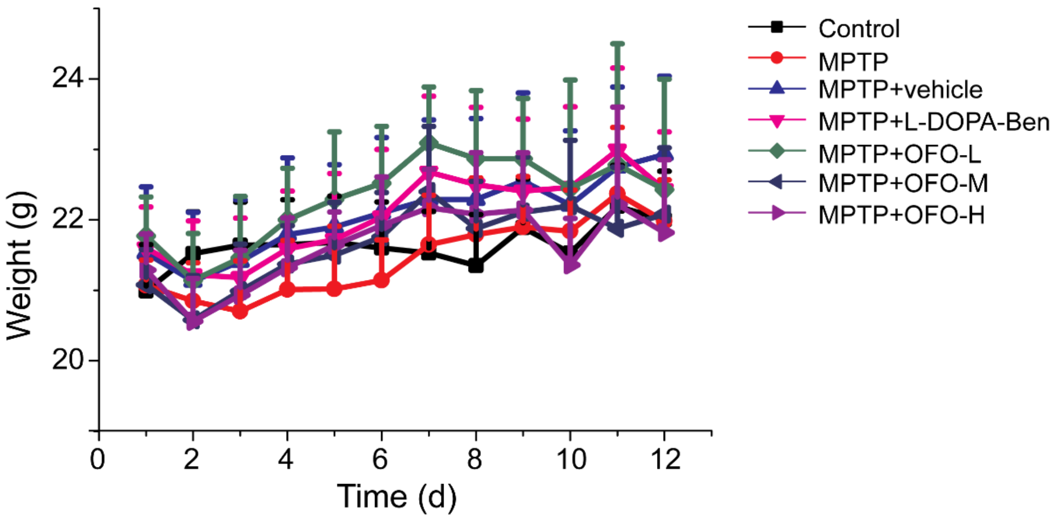

Figure S8. The weight changes during the 12-day prevention of PD.
